# Building stock market resilience through digital transformation: using Google trends to analyze the impact of COVID-19 pandemic

**DOI:** 10.1186/s11782-020-00089-z

**Published:** 2020-09-21

**Authors:** Ding Ding, Chong Guan, Calvin M. L. Chan, Wenting Liu

**Affiliations:** School of Business, Singapore University of Social Sciences, 463 Clementi Rd, Singapore, 599494 Singapore

**Keywords:** Digital transformation, Stock market, Search trends, Market sentiment, 2019 novel coronavirus disease (COVID-19)

## Abstract

As the 2019 novel coronavirus disease (COVID-19) pandemic rages globally, its impact has been felt in the stock markets around the world. Amidst the gloomy economic outlook, certain sectors seem to have survived better than others. This paper aims to investigate the sectors that have performed better even as market sentiment is affected by the pandemic. The daily closing stock prices of a total usable sample of 1,567 firms from 37 sectors are first analyzed using a combination of hierarchical clustering and shape-based distance (SBD) measures. Market sentiment is modeled from Google Trends on the COVID-19 pandemic. This is then analyzed against the time series of daily closing stock prices using augmented vector autoregression (VAR). The empirical results indicate that market sentiment towards the pandemic has significant effects on the stock prices of the sectors. Particularly, the stock price performance across sectors is differentiated by the level of the digital transformation of sectors, with those that are most digitally transformed, showing resilience towards negative market sentiment on the pandemic. This study contributes to the existing literature by incorporating search trends to analyze market sentiment, and by showing that digital transformation moderated the stock market resilience of firms against concern over the COVID-19 outbreak.

## Introduction

The onslaught of the 2019 novel coronavirus disease (COVID-19) pandemic has impacted stock markets worldwide, with the stock prices of many firms seeing unprecedented fall. Many governments around the world imposed a shutdown on their cities in attempts to implement social distancing practices and slow down the spread of the life-threatening virus. Such shutdowns have brought what is classified as non-essential corporeal economic activities close to complete standstill. This has resulted in well-known brands, such as Hertz, JC Penny, and J Crew, filing for bankruptcy, and sparking concerns of large-scale economic recession or even economic collapse (Nicola et al. 2020).

Despite this, amidst the bleak economic outlook, certain sectors appeared to have performed better. Reports have indicated that even as most corporeal economic activities halted, trade and consumption continued to take place online as consumers were spending more time and money online as the virus peaked (Huang et al. 2020). This paper aims to investigate the sectors that have performed better even as market sentiment is affected by the unfolding of the pandemic.

The remainder of the paper is organized as follows. Section 2 presents the literature review, covering three areas on the effect of large-scale unanticipated incidents on the stock market, the association between online search trends and stock market performance, and the digital transformation of firms. The McKinsey Global Institute’s (MGI) Industry Digitalization Framework (Gandhi et al. 2016; Manyika et al. 2016), which is used as the basis to classify the sectors in this study, is also introduced in this section. Section 3 describes the empirical investigation, detailing the research design, as well as data sources and analysis. The results of the analysis are then presented in section 4. Section 5 features the discussion and the implications of the findings for both research and practice.

## Literature review

This paper aims to investigate the sectors that have performed better even as market sentiment is affected by the pandemic. The COVID-19 pandemic is conceptualized as an instance of large-scale unanticipated incidents which include natural disasters such as hurricanes and earthquakes (Lee et al. 2018; Shelor et al. 1992) or major incidents such as aviation accidents and terrorist attacks (Barrett et al. 1987; Cam and Ramiah 2014). Thus, the first sub-section of this literature review is based on the effects of large-scale unanticipated incidents on stock markets.

In studying market sentiment, this paper adopts a novel approach to track it through online search trends. Thus, the second sub-section of this literature review focuses on how online search trends have been employed in studying investors’ behavior and stock market performances.

Further, considering the observation that firms which engaged in digital transformation performed ostensibly better in terms of the financial markets, the third sub-section on the topic is presented.

### Effect of large-scale unanticipated incidents on stock markets

According to the Efficient Market Hypothesis, stock prices are expected to adjust immediately, without any overreaction, when new information is made available to the public. However, in the real world, investors are not always rational. Studies in behavioral finance (Nofsinger 2003) have shown that investors may over-react in the short term when they become extremely pessimistic during downturns or place too much importance on recent events while ignoring historical data. This results in a steep fall in stock prices on adverse news. Previously, various studies have examined investor behavior amidst unprecedented large-scale disasters, such as earthquakes or terrorist attacks (Barrett et al. 1987; Cam and Ramiah 2014; Lee et al. 2018; Shelor et al. 1992). They find significant stock price overreactions, which are consistent with an availability bias known from the behavioral finance literature. This kind of reaction by investors is understandable, given the exceptional damages and the subsequent high attention these events receive from the media.

Similar to other types of natural disasters or major incidents, COVID-19 is an unexpected large-scale incident whose impact on the global economy is manifested profoundly in global financial markets soon after the outbreak of the pandemic. The rapid spreading of the virus and sharp increase in the death tolls have had a large emotional and material impact and are widely regarded as “market-wide shocks,” which has changed the investor perception on the micro- and macro-economic environment of stock markets.

Previously, epidemics like Influenza, Ebola, and Severe Acute Respiratory Syndrome (SARS) have influenced a large number of economies worldwide. All of these epidemics have resulted in severe damage to the economy and loss of lives, e.g., 5% gross domestic product (GDP) of the US was reduced owing to the Influenza (Palese 2004); as result of Ebola, the US encountered a deficit of USD53 billion, and there were more than 11,300 deaths in the world (Hai et al. 2004). The SARS affected more than 8,000 people and caused a 1% drop in China’s GDP and the economic damage of USD54 billion worldwide. However, none of the previous epidemics was as comparable to the recent pandemic COVID-19 in terms of the damage caused to the economy and the number of people affected. By April 19, 2020, COVID-19 has infected more than 2.3 million people and killed at least 163,000 worldwide, and the numbers were still increasing. Meanwhile, the global stock markets confronted an extreme collision in their market values. The market value of the world’s major stock index, such as the Standard & Poor (S&P)500, National Association of Securities Dealers Automated Quotations System (NASDAQ)100, and Nikkei225 dropped nearly 30% since the outbreak of COVID-19. Additionally, the financial volatility index (VIX), also known as “Fear gauge,” has moved to the uppermost level. In contrast, the U.S. 10-year treasury yield index moved down to record low level (Leduc and Liu 2020). The Asian Development Bank (ADB) evaluates that the global cost of COVID-19 can be USD4.1 trillion (ADB 2020). Khurram et al. (in press) believe that the health crisis of COVID-19, causing severe shocks in the stock markets, is likely to produce a global financial crisis.

However, the new digital world has made the COVID-19 pandemic’s impact on the global economy different from that of previous epidemics. The development of digital technologies in the past two decades has changed the way people and firms interact with each other. With the fast-growing Internet users worldwide, online shopping has become one of the most popular online activities, and the emergence of e-business platforms such as Amazon and Alibaba contribute to the rapid growth of online sales. In 2019, e-retail sales accounted for 14.1% of all retail sales worldwide, expected to reach 22% in 2023 (Clement 2019). In the current pandemic-led stock market crash, interestingly, certain industries and firms are able to withstand the adverse effect and turn the crisis into opportunities by providing/using digital solutions to expand their business. Thus, their stock prices are not negatively affected by the market’s downturn. For example, the stock price of Zoom Video Communications, Inc. (the major provider of online conference platforms) has increased from USD70 in early January 2020 to USD150 by the end of March 2020 amidst the market crash.

In the aftermath of the COVID-19 pandemic, it is observed that digital technology and connectivity have emerged as essential tools and an alternative to their physical equivalents in combating the adverse effects of the pandemic and enhancing societal and economic resilience (Atsuko and Karazhantva 2020). Thus, a firm or an industry with a pre-existing digital ecosystem is digitally resilient and can cope with emergencies, and therefore instill investors’ confidence in the company and its stocks. Particularly, we argue that sectors with higher levels of digital transformation remain resilient to the impact of the market sentiment from the COVID-19 pandemic, while sectors that lag across most digital transformation dimensions are among the most negatively affected.

### Online search and financial markets

Most traditional research that investigates the effects of large-scale unanticipated incidents on stock markets has mainly employed intervention analysis (Box and Tiao 1975) and event studies (Mackinlay 1997). The effects of such events are assessed using dummy variables. Liu et al.’s (2019) study on the effects of aviation accidents on financial markets, is an exception, in which they directly measure market sentiment towards critical events, using Google and the Baidu Index searches, and quantify these effects on stock prices of the affected airline. Their results confirm the feasibility of using the search index as a proxy for market sentiment to investigate how critical events affect stock prices. The current research builds upon Liu et al.’s (2019) study in applying a search index as an indication of market sentiment towards a large-scale unanticipated incident, i.e., COVID-19, to study its impact on the stock price performance of firms across sectors. Although using search index as the proxy for market sentiment is still an indirect measure of the impact of the incident, it is a great improvement over the dummy variables used in previous studies because search data is a more accurate and timely reflection of the market sentiment and offers insights into investors’ behaviors.

Search trends capture the collective interest of investors and are thus a reflection of market sentiment and useful indicators for predicting human decision making (Liu et al. 2019; Weng et al. 2018). Search data measure the public’s attention to unexpected events and can serve as a timely marker of investment dynamics. For example, striking patterns shown in Google search trends can be utilized as sources of strategic investor information in predicting stock market movements. Market dynamics can change rapidly, and it can be hard to grasp market sentiment that is evolving in tandem. Internet search trends reveal query trends of significant public interest, almost in real-time. Hence, financial market analysis is based on tracking online behavior, such as Internet search trends. Its application to stock market research has also drawn scholarly attention. Da et al.’s (2011) study is the first application of Google search data in the financial field. They propose a method of measuring investors’ attention to different stocks by referencing Google search trends. Bordino et al. (2012) study the relationship between NASDAQ100 trading volumes and search volumes extracted from Yahoo’s search query logs, and identify a positive correlation between search volume related to a stock on a particular day and its trading volume over the several following days. Vlastakis and Markellos (2012) report that investors seek more information when risk aversion begins to rise. Joseph et al. (2011) use search data as the proxy of investor sentiment and obtain reliable forecasts of abnormal stock returns and trading volumes. Finally, Andrei and Hasler (2014) document the relationships between search data and market fluctuation in the international exchange market.

This research thus extends the above-mentioned literature by investigating the effect of market sentiment associated with COVID-19, derived using Internet search trends, upon the stock market performance of firms across different sectors.

### Digital transformation of firms

Even as the spread of the COVID-19 has led to the stalling of much of the brick-and-mortar based economic activities, reports have shown that digitally-enabled economic activities and business transactions are not only taking place online but have grown during the outbreak (Baig et al. 2020; Huang et al. 2020). Digital transformation, also known as digitalization, is defined as the changes a firm goes through as it starts to use digital technologies to develop a new digitally-enabled business model to create and appropriate more value for the firm (Kane et al. 2019; Liu et al. 2011; Schallmo et al. 2017). Thus, firms that have undergone digital transformation are likely to have a better chance of performing better amidst the pandemic as they are more capable of maintaining some degree of operation and revenue stream for their businesses. Market sentiment towards such firms may thus be stronger and, consequently, translate into more resilient stock prices.

Interest in digital transformation has flourished in recent years, and it has become an important topic in management research as well as for practitioners in the business world. At the macro level, digital transformation has resulted in profound changes that can be seen in the society and different sectors with widespread adoption and use of digital technologies (Agarwal et al. 2010; Majchrzak et al. 2016). At the micro-level, research has indicated that firms must innovate with these technologies by devising “strategies that embrace the implications of digital transformation and drive better operational performance” (Hess et al. 2016).

Digital technology is the building block of digital transformation (Chan et al. 2019), and nine types of such technology have been identified. These include big data and analytics, autonomous robots, simulation, horizontal and vertical system integration, Internet of things (IoT), cybersecurity, cloud computing, additive manufacturing, and augmented reality (Rüßmann et al. 2015). Among these, IoT, cloud computing, big data, and analytics are considered to be key for firms in innovating new service-oriented business models.

Regarding how digital transformation can help firms in staying competitive, technology has always been deemed to be a catalyst for developing a service business orientation (Kowalkowski et al. 2013), and a key resource in managing the different issues that arise from complex product-service systems (Neu and Brown 2005). In particular, the current technological revolution has created opportunities for a service-oriented transformation of the business models of manufacturing firms that are deemed to be important by both business-oriented (e.g., Noventum Service Management 2016) and academic literature (e.g., Coreynen et al. 2017). Technology such as IoT thus offers new opportunities to firms to create innovative and new business models and transform from traditional manufacturing to smart manufacturing where the operations are controlled by artificial intelligence and can be supervised remotely. During the onset of the COVID-19 pandemic as shops and factories are closed due to social distancing regulations, firms that have embarked on digital transformation respond better than those who have not. They conduct some business activities digitally (Blackburn et al. 2020; Huang et al. 2020) instead of a complete standstill.

Recent research from the MGI (Gandhi et al. 2016; Manyika et al. 2016) examines the state of digital transformation across various sectors and finds that there is a large and growing gap across the sectors. It is found that firms that are more advanced in their digital transformation experience better growth in productivity and profit margins. Six categories of sectors are identified, namely:

1. knowledge-intensive sectors that are highly transformed digitally across most dimensions;
2. capital-intensive sectors with the potential to further transform their physical assets digitally;
3. service sectors with a long tail of small firms having room to transform their customer transactions digitally;
4. B2B sectors with the potential to digitally engage and interact with their customers;
5. labor-intensive sectors with the potential to provide digital tools to their workforce;
6. quasi-public and/or highly localized sectors lagging across most dimensions.

**Fig. 1:**
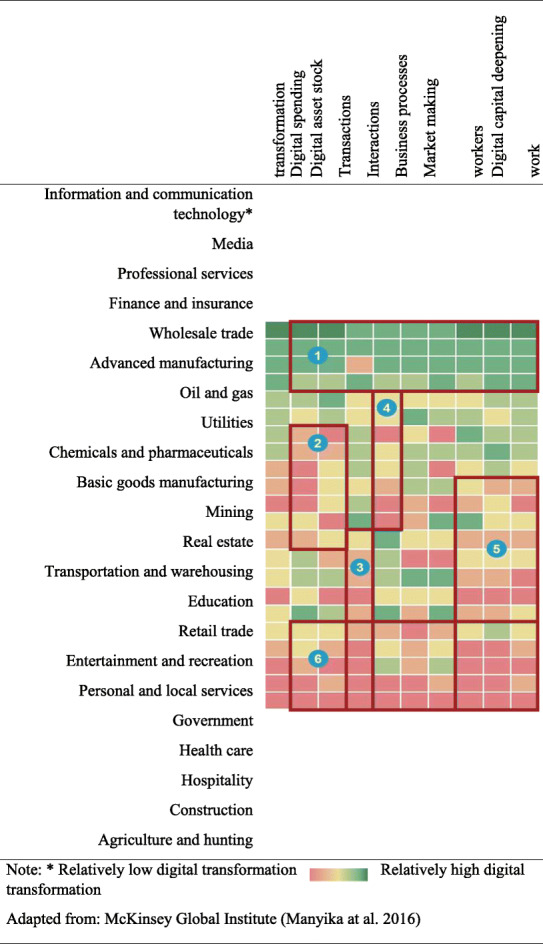
MGI Industry Digitalization Framework

The MGI Industry Digitalization Framework, as illustrated in Fig. 1, shows the level of the digital transformation of the various sectors, and gives a clear picture of where the sectors stand in the development of digital transformation. It combines indicators to show how firms are building digital assets, expanding digital usage, and creating a more digital workforce. This indicates that, along with the information and communication technology sector, media, financial services, and professional services are ahead of the rest of the sectors in terms of digital transformation. By contrast, sectors such as health care, education, personal and local services, hospitality, basic goods manufacturing, and construction are lagging considerably behind. The uneven pace of digital transformation is creating a new digital divide between the digital “haves” and “have-mores” across sectors and among firms. The most highly digitally transformed sectors have posted two to three times higher rates of growth in profit margin, than others, and wages, than the national average. Firms with advanced digital assets and capabilities have generated higher rates of revenue growth and higher return to shareholders. The “have-mores” are not just large firms that dominate one sector. They can also be small, innovative firms or firms whose digital assets enable them to play in multiple sectors.

## Empirical investigation

### Research design

To investigate the sectors that have performed better even as market sentiment is affected by the unfolding of the pandemic, an empirical study was designed based on data from Google search trends and stock prices. The MGI Industry Digitalization Framework was referenced as a basis to organize the sectors.

The study consisted of two phases. Phase 1 utilized hierarchical clustering and shape-based distance (SBD) to validate the groups of firms identified in the MGI Industry Digitalization Framework. It followed similar approaches employed by Sardá-Espinosa (2019) and Paparrizos and Gravano (2017). This analysis identified two distinct clusters, i.e., a *Sensitive Cluster* and a *Resilient Cluster*.

Phase 2 modeled Google search trends and stock price changes jointly in an augmented VAR. In this phase, the analysis compared and contrasted the stock performance of firms across the three levels of digital transformation, i.e., low, medium, and high, based on the MGI Industry Digitalization Framework. The analysis in the two phases built on each other, supporting the notion that market sentiment towards the COVID-19 pandemic as reflected in Google search trends affects the stock prices.

### Data source and analysis

Using data from FactSet, we selected nearly 2000 firms listed on the NASDAQ based on their sectoral information in 2020. NASDAQ is one of the world’s largest stock exchange based on market capitalization. Among the scholarly community, NASDAQ represents one of the most widely investigated stock exchange databases (e.g., Bordino et al. 2012; Weng et al. 2018).

The sectors are selected according to the MGI Industry Digitalization Framework (Gandhi et al. 2016; Manyika et al. 2016). The sample was thus classified into the six categories of sectors as shown in Fig. 1.

From the initial pool, entries with missing data are eliminated. Pharmaceutical and biopharmaceutical companies play a key role on the front lines of the battle against the coronavirus. While global stock markets take a COVID-19 beating, pharmaceutical stocks are generally performing better than those of other industries, with additional investments in the race to develop coronavirus vaccines and therapeutics. Makers of diagnostic test kits, sanitizers, and protective masks have all ramped up to meet the unprecedented demand. As the current research focuses on how digitally transformed sectors show resilience towards negative market sentiment on the pandemic, the pharmaceutical and biopharmaceutical companies have been removed from the sample to avoid confounding effects, as each of these stocks has the potential for considerable gain, whether it is because they are developing a treatment or their products are in greater need amid the outbreak. The final dataset admitted for analysis included 1,568 firms across 37 sectors.

Although the stocks in our sample cover only 36.6% of all the stocks traded on the NASDAQ, they are an economically significant component of the total market as they accounted for 45% of the NASDAQ trading volume in the first quarter of 2020. The range of market capitalization represented in our NASDAQ sample is substantial (ranging between USD 16,280,208 and USD 6999 million). The average market capitalization for the sampled stocks is USD8,587 million. The sample period (all trading days from early January 2020 to early April 2020) is chosen because it allows a reasonable amount of time lag for the COVID-19 outbreak to generate public attention and stir market sentiment. Additionally, the current research focuses on how sectoral digital transformation mitigates the effect of negative events on the stock market. We are more interested in the period from the outbreak of the COVID-19 to the eventual stock market crash (the affected period). There might be other potential confounding factors coming into play during the recovery period. For example, the U.S. coronavirus relief funds, established under the Coronavirus Aid, Relief, and Economic Security (CARES) Act, were announced in early April 2020. The stock market is likely to respond to such information on economic support. The chosen sample period ends when the CARES Act was announced, minimizing the potential confounding effect of government support and intervention on the stock market. For every entry, three data fields are obtained, namely, the daily price change, trading volume, and market cap. These data are used in the models in both Phase 1 and Phase 2. Table 1 provides the descriptive statistics of the firms.

**Table 1:** Descriptive statistics by sector

Sector	Average market cap (USD Mil)	Average daily trading volume (Mil)	Average daily price change (%)	Sample size
Media conglomerates	223,611.95	18.17	− 0.51%	1
Internet retail	70,518.85	3.52	−0.14%	23
Managed health care	60,663.37	2.57	−0.16%	8
Major telecommunications	52,500.63	7.34	−0.05%	10
Food retail	35,804.03	2.58	0.35%	10
Telecommunications equipment	29,147.66	2.92	−0.29%	50
Internet software/services	27,785.03	2.25	−0.40%	93
Electric utilities	17,336.85	2.58	−0.21%	51
Wireless telecommunications	16,942.21	2.01	1.07%	18
Data processing services	12,887.17	1.33	−0.43%	40
Insurance brokers/services	10,053.82	0.69	−0.31%	14
Oil and gas pipelines	9849.43	5.53	−1.30%	11
Movies/entertainment	8878.23	1.12	−0.72%	28
Hotels/resorts/cruise lines	8444.96	4.59	−1.07%	18
Chemicals: major diversified	8322.61	2.20	−0.67%	9
Information technology services	8139.05	1.34	−0.26%	109
Chemicals: specialty	7458.86	0.90	−0.52%	44
Trucks/construction/farm machinery	6352.26	0.77	−0.61%	35
Real estate investment trusts	5922.43	1.89	−0.65%	207
Computer communications	5286.76	0.65	−0.05%	13
Apparel/footwear retail	5253.82	2.10	−1.02%	37
Oil and gas production	4484.33	7.14	−0.29%	70
Chemicals: agricultural	4298.97	1.39	−0.49%	14
Construction materials	3859.77	0.55	−0.87%	10
Water utilities	3688.86	0.36	0.03%	12
Agricultural commodities/milling	2938.48	2.06	−0.42%	19
Financial conglomerates	2778.31	0.50	−0.24%	109
Biotechnology	2175.75	1.31	−0.01%	285
Wholesale distributors	2219.28	0.53	−0.54%	45
Advertising/marketing services	2058.79	1.77	−0.80%	22
Miscellaneous manufacturing	1891.37	0.52	−0.53%	14
Real estate development	1795.77	0.55	−0.57%	43
Engineering and construction	1633.11	0.58	−0.54%	32
Specialty telecommunications	1526.04	1.57	0.14%	19
Personnel services	1124.66	0.26	−0.60%	25
Other transportation	1058.49	0.40	−0.70%	7
Coal	745.55	0.55	−0.59%	13

**Fig. 2:**
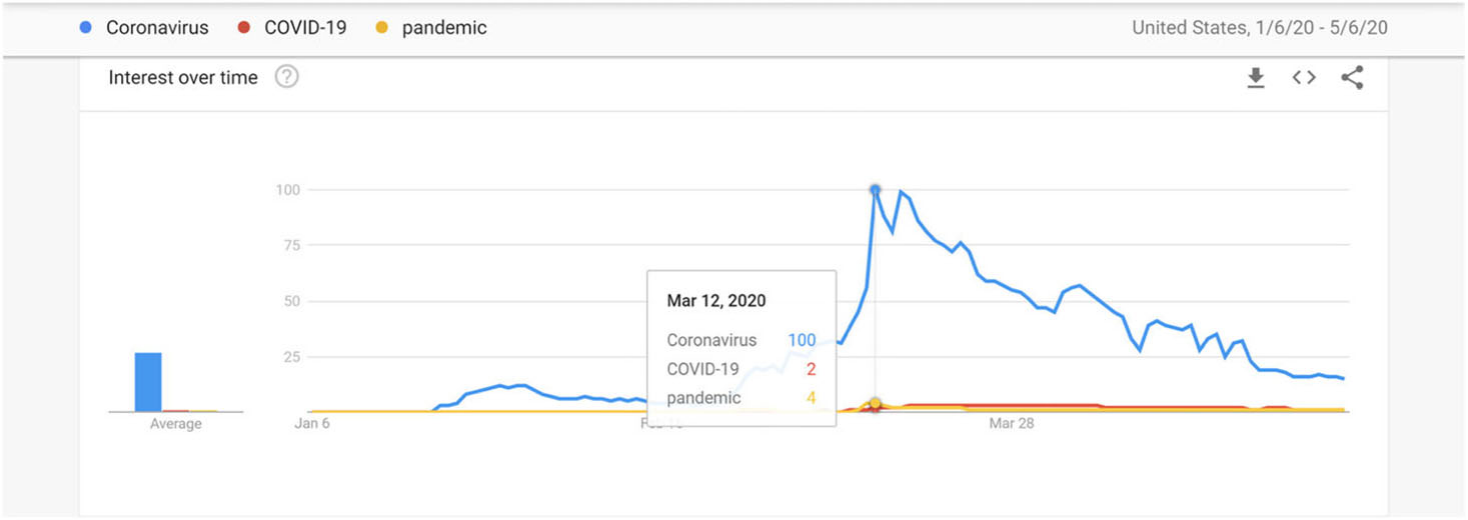
Keywords in Google searches related to COVID-19

In Phase 2, the search variable is operationalized as an “interest over time,” measured on a relative scale of 0–100, with peak popularity coinciding with a value of 100. While Google’s indicator of interest, over time, has been used as a source of data in numerous studies (e.g., Da et al. 2011), how these search behaviors change during the pandemic has not been widely investigated hitherto. Various event-related keywords are first explored. For example, the search for keywords such as “COVID-19” and “pandemic” are compared with those for “coronavirus.” However, Google trends reveal that none of these search terms matches the trend of search interest on coronavirus (see Fig. 2). Thus, coronavirus is adopted as the most relevant event search keyword. The daily Google trends of the keyword coronavirus during this period is thus matched against the stock prices of the selected firms.

## Results

### Phase 1 results

The main innovation of the methodology employed in this study is the shift from comparing descriptive indicators among firms to clustering of time series indicators based on stock performance. The clustering of firms is based on how their market capitalizations change along with market sentiment towards the pandemic. This allows firms that perform similarly to be identified and also to discover the common attributes shared among the proximate firms. The identified clusters are further explored to understand the underlying reasons for the common patterns they exhibit. Liao (2005) provides a complete view of the available time series clustering approaches. Three major categories of clustering techniques have been adapted to cope with time-series data. It consists of partitioning, hierarchical, and model-based methods. According to Liao (2005), the most available time-series clustering approaches are in general variations of k-means or hierarchical clustering with a range of specified dissimilarity functions designed for the problem. A combination of clustering method and dissimilarity measures is deemed feasible and appropriate to analyze our dataset and address the research problem. Common methods like k-means, fuzzy c-means, and self-organizing maps work better with time series of equal length, which means all data points to be filled across the same period. However, hierarchical clustering applies to time series with unequal length when an appropriate distance measure like Dynamic Time Warping (DTW) distance is chosen to compute dissimilarity (Paparrizos and Gravano 2017). This combination is more applicable to our dataset because not all the firms traded on the same days during the observed period. Thus, this study uses a combination of hierarchical clustering and SBD, which is a faster alternative to DTW (Sardá-Espinosa 2019).

The hierarchical clustering method is a tree-based technique that groups objects into a tree hierarchy of clusters in an agglomerative or divisive manner (Hastie et al. 2009). The agglomerative method treats each object as an individual cluster and iteratively groups clusters into larger clusters based on the pairwise similarity measures. It only stops when all objects fall into a single cluster or meet certain criteria, e.g., reaching the maximum number of clusters. The divisive method starts from a single big cluster that contains all objects and splits the cluster iteratively until each object belongs to an individual cluster. The SBD was proposed by Paparrizos and Gravano (2017) based on the normalized cross-correlation (NCC). It ranges from 0 to 2, with 0 representing perfect similarity. The distance can be obtained from Eq. (1). We adopt SBD to be the distance measure and the shape extraction to define centroid with a z-normalization preprocessing (Sardá-Espinosa 2019).

1 $$ SBD\left(x,y\right)=1-\frac{\max \left( NCCc\left(x,y\right)\right)}{{\left\Vert x\right\Vert}_2{\left\Vert y\right\Vert}_2} $$

Hierarchical clustering requires a decision of the optimal number of clusters, which can be subjective. It is recommended to evaluate the clustering performance based on the cluster validity indices (CVIs) (Arbelaitz et al. 2013; Lei et al. 2017). Among the commonly used crisp CVIs, internal indices are to evaluate cluster purity, and the external CVIs are used to cross-check the results with a known correct clustering result. A summary of the common CVIs and the evaluation criteria is shown in Table 2.

**Table 2:** Cluster validity indices

CVIs	Literature	Evaluation criteria
Silhouette (Sil)	Rousseeuw (1987)	Maximized
Score Function (SF)	Saitta et al. (2007)	Maximized
Calinski-Harabasz (CH)	Arbelaitz et al. (2013)	Maximized
Davies-Bouldin (DB)	Arbelaitz et al. (2013)	Minimized
Modified Davies-Bouldin (DBstar)	Kim and Ramakrishna (2005)	Minimized
COP	Arbelaitz et al. (2013)	Minimized

Following the above-stated method, the data are tested across different numbers of clusters, ranging from two to seven clusters via an R package proposed by Montero and Vilar (2014). The results are shown in Table 3. A majority of the CVIs indicate that the optimal number of clusters is at two. There are 742 firms in Cluster 1 and 1154 firms in Cluster 2.

**Table 3:** Results of CVIs

CVIs	K = 2	K = 3	K = 4	K = 5	K = 6	K = 7
Sil	7.15E-01	6.96E-01	6.89E-01	6.83E-01	6.74E-01	6.70E-01
SF	7.01E-01	5.84E-01	4.52E-01	4.42E-01	4.64E-01	4.15E-01
CH	1.55E+ 04	7.78E+ 03	5.43E+ 03	4.09E+ 03	3.28E+ 03	2.78E+ 03
DB	2.95E-01	4.29E-01	7.28E-01	6.65E-01	6.11E-01	6.25E-01
DBstar	2.95E-01	4.66E-01	7.65E-01	7.14E-01	6.75E-01	6.73E-01
COP	1.34E-01	1.33E-01	1.23E-01	1.23E-01	1.22E-01	1.20E-01

By analyzing the extracted centroid, the times series patterns of the two clusters are then examined. For firms in Cluster 1, it is observed that a steeper declining trend of market capitalization occurs amid the COVID-19 outbreak. This is thus termed as the *Sensitive Cluster*. That is, these firms are likely to be more sensitive to the pandemic. In comparison, firms in Cluster 2 are found to be more resilient to the pandemic with a flatter market capitalization curve both pre and post the declaration of the pandemic by the World Health Organization (WHO) on March 11, 2020. Thus, this cluster is named as the *Resilient Cluster.*

The firms are cross-tabulated with percentages of their market capitalization decline under the *Sensitive Cluster* and the *Resilient Cluster*, with a high, medium, and low level of digital transformation following the MGI Industry Digitalization Framework, to answer the research question and to validate the sectoring. To further simplify the research model and to enable in-depth investigation in Phase 2, Categories 2 to 5 in the original MGI Industry Digitalization Framework, which represent sectors with the potential to transform digitally further, are merged into one group of firms with a medium level of digital transformation. Firms under the most digital sectors (Category 1) and the most lagging sectors (Category 6) are left unchanged. It is then analyzed to assess if the level of digital transformation is associated with the two identified clusters. A descriptive summary of the sensitive and resilient clusters can be found in Table 4.

**Table 4:** Summary of performance

Level of digital transformation	Sector	Group	Sensitive	Resilient
High	Biotechnology^c^;	Group 1	32%^b^	68%^b^
	Information technology services;			
	Financial conglomerates;			
	Internet software/services;			
	Telecommunications equipment;			
	Data processing services;			
	Personnel services;			
	Advertising/marketing services;			
	Specialty telecommunications;			
	Wireless telecommunications			
Medium	Real estate investment trusts;	Group 2	52%^a^	48%^a^
	Pharmaceuticals: major^d^;			
	Pharmaceuticals: other^d^;			
	Oil and gas production;			
	Electric utilities;			
	Wholesale distributors;			
	Chemicals: specialty;			
	Real estate development;			
	Apparel/footwear retail;			
	Trucks/construction/farm machinery			
Low	Agricultural commodities/milling;	Group 3	60%	40%
	Hotels/resorts/cruise lines;			
	Managed health care			

^a^significant at 1% level, ^b^significant at 5% level; ^c^85 pharmaceutical and biopharmaceutical companies were removed from the Biotechnology sector; ^d^244 pharmaceutical companies were removed

Consistent with expectation, more than two-thirds of the firms (68%) within the most digitally transformed sectors, such as telecommunications equipment, personal services, financial conglomerates, and advertising/marketing services, fall under the *Resilient Cluste*. The majority of the firms in the lowest rung (60%), including agriculture, hotels, and healthcare, fall under the *Sensitive Cluster.* Firms with mid-level of sectoral digital transformation fall in between, with about half of the firms (48%) being in the *Resilient Cluster*. Additionally, Pearson *χ*^2^ statistic is used to test the two-way associations with frequencies in the cells (*χ*^2^ (1) =16.667, *p* < 0.05). The results provide some directional support to the relative impact of digital transformation. It also validates the relevance of the level of digital transformation (high, medium, and low) as a grouping variable to be used in the analysis in Phase 2.

### Phase 2 results

**Fig. 3:**
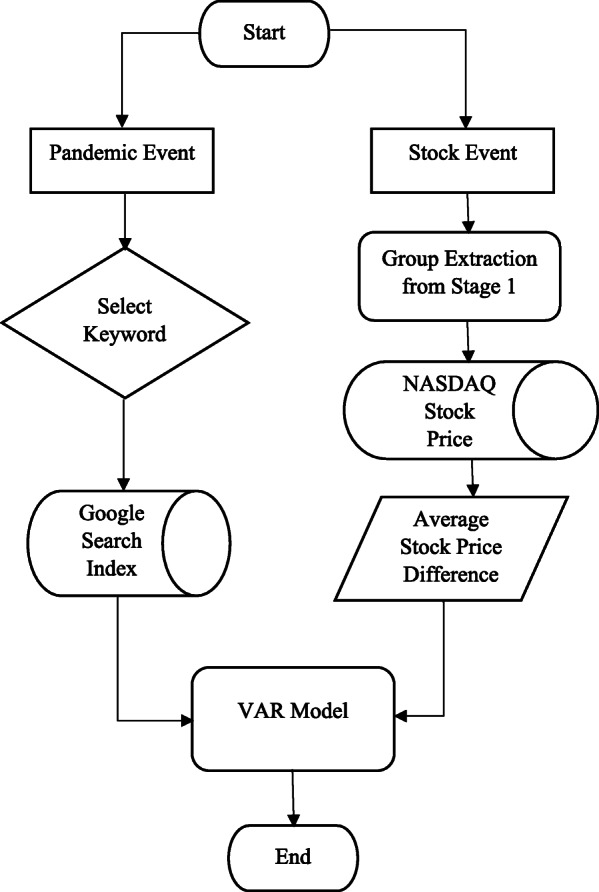
Analysis process: Phase 2

Google trends index represents the search volume of keywords by Google users, and it serves to indicate market sentiment and the public’s attention to an event or incident (Liu et al. 2019). We apply the Google search index on “coronavirus” as a variable of interest. The other set of variables are the average daily stock price differences between the three groups of firms with a high, medium, and low level of digital transformation derived from Phase 1. We use a VAR model (e.g., Deng et al. 2018; Liu et al. 2019; Tetlock 2007) to test the mutual causality relationship between Google trends and stock price differences. One direction is to estimate the effects of the Google search trends on the stock price fluctuations for each group of firms. The other direction is to test if the stock price differences induce or reduce public attention on the incident. We also investigate the timing and duration of such impacts. An overview of the Phase 2 analysis is shown in Fig. 3. The algorithm is implemented via R.

Firstly, it examines the relationship between the Google search index and the stock price fluctuations, including the direction and the timing of the effects through Granger causality and VAR model. From the plotted pair of variables, the stock price change, and the adjusted Google search index for coronavirus (the original index/100), a negatively correlated movement of the two variables for the three groups of firms is observed. Stock prices move downwards when the search for coronavirus increased. We do not test correlation, given that it does not necessarily reflect causality, and Granger causality is used to test the leading relationship between these two variables. However, the typical Granger causality test cannot be relied on when one or both time series are non-stationary, which could lead to spurious causality (He and Maekawa 2001). Thus, an Augmented Dickey-Fuller (ADF) test is employed. Besides, a Kwiatkowski-Phillips-Schmidt-Shin (KPSS) test in which the null hypothesis is stationarity is also conducted as a cross-check.

Additionally, the worldwide plunge in stock markets in early March 2020 could be partially attributed to the worst oil price collapse after an all-out price war erupted among the world’s biggest producers. The oil price war could further spark market anxiety, adding to virus worries. To factor in the impact of public attention on oil price, which fluctuated during the research period, we also include the Google search index for “oil”^1^as a control variable, which might exert an impact on stock performance. Table 5 summarizes the ADF and KPSS statistics for the Google search index for coronavirus and oil and the average day-on-day stock price changes for each group of firms. This reveals that the first-order difference of these variables complies with the stationarity assumption required for Granger causality. Although our research interest is to investigate the causality relationship between Google search index for coronavirus and the average day-on-day stock price changes, we still test for the Google search index for oil to check the stationarity of the time series inserted into the model. This determines the order of integration and time-series models following the Toda-Yamamoto (TY) (Toda and Yamamoto 1995) procedure.

2 $$ {Y}_t={a}_0+{a}_1{Y}_{t-1}+..\dots +{a}_p{Y}_{t-p}+{b}_1{X}_{t-1}+..\dots +{b}_p{X}_{t-p}+{e}_1{Z}_{t-1}+..\dots +{e}_p{Z}_{t-p}+{u}_{t.} $$

3 $$ {X}_t={c}_0+{c}_1{X}_{t-1}+..\dots +{c}_p{X}_{t-p}+{d}_1{Y}_{t-1}+..\dots +{d}_p{Y}_{t-p}\kern0.5em +{v}_{t.} $$

**Table 5:** Tests of ADF and KPSS

Variable	Definition	ADF statistics	KPSS statistics
*G*_*t*1_	Google search index: coronavirus	−2.3385	1.2101^a^
*D* (*G*_*t*1_)	Difference of Google search index: coronavirus	−3.5195^c^	0.0991
*O*_*t*1_	Google search index: oil	−3.3784	0.7009^b^
*D* (*O*_*t*1_)	Difference of Google search index: oil	−5.6083^b^	0.0439
*P*_*t*1_	Day-on-day average price change for Group 1	−2.9110	0.1335
*D* (*P*_*t*1_)	Difference of day-on-day average price change for Group 1	−4.8725^b^	0.0752
*P*_*t*2_	Day-on-day average price change for Group 2	−3.0845	0.1479
*D* (*P*_*t*2_)	Difference of day-on-day average price change for Group 2	−5.8872^b^	0.0687
*P*_*t*3_	Day-on-day average price change for Group 3	−3.1117	0.1205
*D* (*P*_*t*3_)	Difference of day-on-day average price change for Group 3	−5.0339^c^	0.0864

^a^significant at 0.1% level; ^b^significant at 1% level; ^c^significant at 5% level

In Eqs. (2) and (3), *Y*_*t*_ represents the average day-on-day stock price change in percentage, *X*_*t*_ and *Z*_*t*_ represent the Google search index of the keyword coronavirus and oil, respectively. Meanwhile, *p* is the lag order, and *a*_*p*_*, b*_*p*_, *c*_*p*_, *d*_*p*_, *e*_*p*_*,* are the coefficients of *Y*_*t-p*_*, X*_*t-p*_, and Z_*t-p.*_ Additionally, *a*_0_ and *c*_0_ are the constant terms and *u*_*t*_ and *v*_*t*_ are the error terms. The null hypothesis for Eq. (2) is H_0_: *b*_1_ = *b*_2_ = ... = *b*_*p*_ = 0, and the alternative hypothesis is H_A_: Not H_0_. When H_0_ is rejected, *X* is the Granger-cause of *Y*. Similarly for Eq. (3), H_0_: *d*_1_ = *d*_2_ = ... = *d*_*p*_ = 0, against H_A_: Not H_0_, is to test the hypothesis that *Y* does not Granger-cause *X*. To interpret the equations, we are to test if *Y* could be better predicted by the histories of its own and *X* than its history. If H_0_ could be rejected, then it implies a Granger causality. We did not explicitly build the hypothesis for effect between *Y*_*t*_ and *Z*_*t*_, because the research question is to investigate whether there is a causal relationship between market performance and public attention to coronavirus.

As shown in Table 5, the first-order difference of variables *X*, *Y*, and *Z* removes the unit root. Thus, the maximum order of integration is set to be 1, denoted by I (1). As a next step, the VAR model is set up using the levels of the data without differencing and determining the appropriate lag length for the variables *X* and *Y*. Based on the information criteria of Akaike Information Criterion Hannan Quinn, Schwarz Criterion, and Final Prediction Error, it is decided that six lags are used in the analyses that followed. As noted by Toda and Yamamoto (1995), the advantage of the TY method is to save the co-integration test and prevent pretest bias. However, there is a need to ensure that the VAR model is specified in a way that there is no serial correlation in the residual value. From the results of a Portmanteau test controlling for dynamic stability, it is observed that Lag 6 removes residual serial autocorrelation for all three groups.

After carrying out the tests for misspecification, the VAR model with Lag 6 is chosen, and one additional lag into each variable is added to Eqs. (2) and (3), given the maximum order of integration I(1). Therefore, an augmented VAR model for Eqs. (2) and (3) is constructed, respectively. This is followed by a Wald test, whereby the hypotheses that the coefficients of the first six lagged values of *X* in Eq. (2) and the coefficients of the first six lagged values of *Y* in Eq. (3) are 0 are tested. The reason not to include the coefficient of the 7th lag is that the additional lagged value is to fix the asymptotic so that the Wald test statistics would be under the null hypothesis that it follows asymptotical *chi*-square distribution. Rejection of the null hypothesis of the Wald test implies a Granger causality. The results are shown in Table 6.

**Table 6:** Test of Granger causality

Null hypothesis	Degree of freedom	*Chi*-square value	Probability
Group 1			
G does not Granger-cause P	6	22.0	0.0012^a^
P does not Granger-cause G	6	22.1	0.0012^a^
Group 2			
G does not Granger-cause P	6	20.9	0.0019^a^
P does not Granger-cause G	6	16.3	0.0120^b^
Group 3			
G does not Granger-cause P	6	19.7	0.0031^a^
P does not Granger-cause G	6	6.8	0.34

^a^significant at 1% level; ^b^significant at 5% level

Table 6 shows that Google search trends affect the stock price fluctuation of firms in all three groups, which confirms our earlier prediction. The stock price changes of firms in Groups 1 and 2 also affect Google search trends, but not Group 3. These results indicate that the changes in stock prices for firms in Groups 1 and 2 can trigger fluctuations in market sentiment. However, the magnitude and the directions of such impact await further investigation from the augmented VAR models, summarized in Tables 7 and 8.

**Table 7:** The results of the augmented VAR estimation: Eq. (2)

Variable	Group 1	Variable	Group 2	Variable	Group 3
*G*_*t*1*–*1_	−0.0029	*G*_*t*2*–*1_	− 0.0040^b^	*G*_*t*3*–*1_	−0.0350^c^
*P*_*t*1*–*1_	−0.1325	*P*_*t*2*–*1_	−0.1430	*P*_*t*3*–*1_	−0.2340
*O*_*t*1*–*1_	0.0009	*O*_*t*2*–*1_	0.0017^c^	*O*_*t*3*–*1_	0.0009
*G*_*t*1*–*2_	0.0019^c^	*G*_*t*2*–*2_	0.0013	*G*_*t*3*–*2_	0.0019
*P*_*t*1*–*2_	0.2694	*P*_*t*2*–*2_	0.3039	*P*_*t*3*–*2_	0.2327
*O*_*t*1*–*2_	−0.0001	*O*_*t*2*–*2_	− 0.0000	*O*_*t*3*–*2_	− 0.0000
*G*_*t*1*–*3_	0.0003	*G*_*t*2*–*3_	0.0016	*G*_*t*3*–*3_	−0.0001
*P*_*t*1*–*3_	0.2626	*P*_*t*2*–*3_	0.2960^c^	*P*_*t*3*–*3_	−0.2918^c^
*O*_*t*1*–*3_	−0.0019	*O*_*t*2*–*3_	−0.2370^c^	*O*_*t*3*–*3_	0.0024
*G*_*t*1*–*4_	−0.0004	*G*_*t*2*–*4_	0.0002	*G*_*t*3*–*4_	0.0004
*P*_*t*1*–*4_	−0.2677	*P*_*t*2*–*4_	−0.1994	*P*_*t*3*–*4_	−0.0454
*O*_*t*1*–*4_	0.0044^a^	*O*_*t*2*–*4_	0.0071^b^	*O*_*t*3*–*4_	0.0061^a^
*G*_*t*1*–*5_	−0.0012	*G*_*t*2*–*5_	−0.0015	*G*_*t*3*–*5_	−0.0021^c^
*P*_*t*1*–*5_	−0.0454^c^	*P*_*t*2*–*5_	−0.3344^c^	*P*_*t*3*–*5_	−0.3233^c^
*O*_*t*1*–*5_	−0.0031^c^	*O*_*t*2*–*5_	−0.0033	*O*_*t*3*–*5_	0.0028
*G*_*t*1*–*6_	0.0013	*G*_*t*2*–*6_	0.0016	*G*_*t*3*–*6_	0.0033^c^
*P*_*t*1*–*6_	−0.4094^c^	*P*_*t*2*–*6_	−0.4133^c^	*P*_*t*3*–*6_	−0.4564^b^
*O*_*t*1*–*6_	0.0017	*O*_*t*2*–*6_	0.0001	*O*_*t*3*–*6_	0.0009
*G*_*t*1*–*7_	0.0006	*G*_*t*2*–*7_	0.0001	*G*_*t*3*–*7_	−0.0002
*P*_*t*1*–*7_	0.2309	*P*_*t*2*–*7_	0.1574	*P*_*t*3*–*7_	0.0640
*O*_*t*1*–*7_	−0.0001	*O*_*t*2*–*7_	0.0000	*O*_*t*3*–*7_	−0.0006

^a^significant at 0.1% level; ^b^significant at 1% level; ^c^significant at 5% level

**Table 8:** The results of the augmented VAR estimation: Eq. (3)

Variable	Group 1	Variable	Group 2	Variable	Group 3
*G*_*t*1–1_	1.0419^a^	*G*_*t*2–1_	0.9475^a^	*G*_*t*3–1_	0.8572^a^
*P*_*t*1–1_	22.1816	*P*_*t*2–1_	−2.5867	*P*_*t*3–1_	−7.5560
*G*_*t*1–2_	−0.4049	*G*_*t*2–2_	−0.3738	*G*_*t*3–2_	−0.3429
*P*_*t*1–2_	0.83528	*P*_*t*2–2_	−15.3009	*P*_*t*3–2_	−19.4786
*G*_*t*1–3_	0.2575	*G*_*t*2–3_	0.1294	*G*_*t*3–3_	0.2056
*P*_*t*1–3_	14.0258	*P*_*t*2–3_	16.0701	*P*_*t*3–3_	20.9271
*G*_*t*1–4_	−0.2058	*G*_*t*2–4_	−0.1458	*G*_*t*3–4_	−0.0987
*P*_*t*1–4_	−32.8371	*P*_*t*2–4_	−14.3280	*P*_*t*3–4_	−17.7680
*G*_*t*1–5_	0.5034^c^	*G*_*t*2–5_	0.5085^c^	*G*_*t*3–5_	0.4119^c^
*P*_*t*1–5_	58.729	*P*_*t*2–5_	19.7339	*P*_*t*3–5_	23.3926
*G*_*t*1–6_	−0.4226^c^	*G*_*t*2–6_	−0.4061^c^	*G*_*t*3–6_	−0.4240^c^
*P*_*t*1–6_	4.2763	*P*_*t*2–6_	−30.5661	*P*_*t*3–6_	−31.6223
*G*_*t*1–7_	−0.0357	*G*_*t*2–7_	−0.0076	*G*_*t*3–7_	0.0390
*P*_*t*1–7_	−63.3119^b^	*P*_*t*2–7_	−62.1916^b^	*P*_*t*3–7_	−50.3626^b^

^a^significant at 0.1% level; ^b^significant at 1% level; ^c^significant at 5% level

As the focus is on the impact that Google searches impose on stock price changes and also to differentiate the impact across the different groups, a detailed investigation into the results of Eq. (2) is then conducted. Consistent with our prediction, Google search trends are positively related to the stock price changes of Group 1, lagged by two periods (2 days). On the other hand, results also show that Google search trends have a one-period lagged negative impact on the stock price changes of Groups 2 and 3. In other words, the relationship between Google search trends and the stock performance of Group 1 rather different than the other two groups. Google search trends can cause negative changes in the stock performance of firms in Groups 2 and 3, with a shorter time lag. In sum, there is strong evidence to show that the digital transformation of firms mitigates the negative impact of market sentiment due to large-scale unanticipated incidents on stock performance. Furthermore, as stock prices dip amid the coronavirus pandemic, firms with mid to high level of digital transformation have out-performed others.

The results of Eq. (3) examine the impact of stock price changes for each group of firms on Google search trends. As indicated in Table 8, it is observed that stock price changes trigger changes in the Google search trend in the opposite direction. Stock price increase in Group 1 will drive a decline of Google search with a lag of seven periods. Similar relationships are observed in Groups 2 and 3. That is, a decline in stock price during the coronavirus outbreak could be easily attributed to the pandemic by the investors, which may further arouse market-wide concerns over the pandemic. Such concerns over the spread of COVID-19 and economic outlook can translate into a higher Google search volume. The impact on Google search trends is strongest for firms in Group1 and weakest for firms in Group 3, with Group 2 falling in the middle.

## Discussion

### Contributions to knowledge and methodology

This study aims to investigate the sectors that have performed better even as market sentiment is affected by the COVID-19 pandemic. Market sentiment on the COVID-19 is modeled with Google search trends. The sectors are organized according to the MGI Industry Digitalization Framework. Performance is quantified by the stock prices of firms in the sectors. The analysis is carried out over two phases.

In the first phase, a three-group model on how stock prices adjust to market sentiment towards the sudden emergence of the COVID-19 pandemic is established. The stock prices of a majority of firms across sectors with a higher level of digital transformation are found to have remained resilient to the impact of market sentiment, while sectors that lag across most digital transformation dimensions are among the most negatively affected.

In the second phase , market sentiment on the COVID-19 pandemic, as reflected in Google search trends, is affirmed to be a predictor of stock performance. There is also further evidence to show that the digital transformation of firms mitigates the negative impact of market sentiment induced by large-scale unanticipated incidents. As the stock price of firms dips amid the coronavirus pandemic, firms with mid to high level of digital transformation have out-performed others. Analysis in the second phase also suggests that a rise in stock price can affect market sentiment.

In answering “what are the sectors that have performed better even as market sentiment is affected by the pandemic,” the analysis reveals that sectors with a higher degree of digital transformation have performed better. Thus, during the COVID-19 pandemic, the market has reacted more favorably towards firms and sectors with a higher level of digital transformation and more negatively towards the laggards. An explanation for this is thus offered.

The spread of the COVID-19 virus has led governments around the world to shut down their cities to slow down the rate and magnitude at which the pandemic is developing. Even as such shutdowns have brought many corporeal economic activities to a near-complete standstill, consumer purchases and even trade not only continue online, but they have increased considerably. Demand for Internet bandwidth as well as portable computing devices such as laptops and tablets also intensify as many people switch to working and learning online. Many are also predicting the emergence of a post-COVID-19 period, where digital transformation will be prominent. Given these, the markets may have given eminence and greater confidence to firms and sectors with a higher level of digital transformation as they are positioned better to sustain operations not only amid the pandemic, but also to recover faster in the post-COVID-19 period. Thus, sectors with a higher level of digital transformation have been more resilient in their performance and also performed better relative to sectors with lower levels of digital transformation.

Further, this study contributes to understanding the role of digital transformation in the firms’ stock market performance, particularly due to large-scale unanticipated incidents such as a pandemic. While previous research has investigated whether the use of technology can improve organizational performance, this research is among the first to demonstrate how digital transformation may give rise to stock market performance. This suggests that digital transformation is an important factor for investors.

The findings from this study also suggest that investors consider recent stock prices when conducting further online search about COVID-19, and such search behaviors will drive changes in stock prices. However, the direction, magnitude, and timing of such effects depend on the level of digital transformation.

Regarding the research method, this study demonstrates the feasibility of using a search trend index to model market sentiment, especially when investigating how large-scale anticipated incidents can affect the stock prices for a large number of firms or across sectors. This presents researchers with another approach to assess market sentiment and expands the research arsenal to include search indexes such as Google trends, which are readily available for use.

### Limitations and future research

This paper has limitations that offer opportunities for future research. First, our study examines how sectoral digital transformation mitigates the effect of market sentiment on negative events (i.e., COVID-19) on the stock market, focusing on the period from the outbreak of the COVID-19 to the eventual stock market crash. Since early April 2020, much of the stock markets’ main indices have regained much of their lost territory for the year. The aggressive stimulus packages roll out by central banks and governments to boost growth, such as the U.S. coronavirus relief funds announced around early April 2020, have fueled recovery. Such confounding effects of government support and intervention on the stock market are difficult to account for in the current research model. Future research can look into how the intervention of governments and support of central banks restore investors’ confidence and accelerate the recovery phase of the market cycle.

The reopening of businesses and economies is also underpinning market optimism. It is plausible that sectoral digital transformation can also influence how firms perform during the recovery period. Therefore, a possible extension of our research is to investigate how digital transformation moderates the speed and extent of the stock market rebound, as it is of interest to both firms and investors. Firms in the most digitally advanced sectors are likely to be better positioned for the recovery and the post-pandemic period.

Second, the literature on the state of digital transformation in sectors suggests a large and growing gap between companies within the sectors (Manyika et al. 2016). We do not investigate the within sector relative differences in this study. Future research may replicate this research by comparing the most digitally transformed firms within each sector with the less digitally transformed one, under a market environment heavily affected by the pandemic.

Lastly, this paper does not investigate the causes behind the growing gap between sectors in digital transformation. Future research may try to uncover how the different conditions lead to a widening gap between the “haves” and the “have-mores”: companies and sectors that are using their digital capabilities far more than others to innovate and transform how they operate.

## Data Availability

Data on stock market prices and Google Search Trend are available from the corresponding author upon request.

## Abbreviations

COVID-19: Disease caused by the coronavirus
2019-nCoV: Coronavirus
SBD: Shape based distance
VAR: Vector Auto Regression
MGI: McKinsey Global Institute
SARS: Severe Acute Respiratory Syndrome
GDP: Gross domestic product
US: United States
S&P: Standard & Poor
NASDAQ: National Association of Securities Dealers Automated Quotations System
Nikkei: Tokyo Stock Exchange
VIX: Volatility index
ADB: Asian Development Bank
IoT: Internet of things
CARES: Coronavirus Aid, Relief, and Economic Security
DTW: Dynamic Time Warping
NCC: Normalized cross-correlation
CVI: Cluster validity indices
WHO: World Health Organization
ADF test: Augmented Dickey-Fuller test
KPSS test: Kwiatkowski-Phillips-Schmidt-Shin test

## Footnotes

1 Various event-related keywords are first explored. For example, the search for keywords such as “oil price,” “petroleum” and “oil war” are compared with those for “oil.” Google trends reveal that none of these search terms matches the trend of search interest on “oil.” Thus, Google search index for “oil” is included as a covariate.
